# A review of approaches to improve participation of culturally and linguistically diverse populations in clinical trials

**DOI:** 10.1186/s13063-016-1384-3

**Published:** 2016-05-26

**Authors:** Jo-anne Hughson, Robyn Woodward-Kron, Anna Parker, John Hajek, Agnese Bresin, Ute Knoch, Tuong Phan, David Story

**Affiliations:** School of Languages and Linguistics, The University of Melbourne, Parkville, VIC 3010 Australia; Department of Medical Education, Melbourne Medical School, University of Melbourne, Parkville VIC, 3010 Australia; Anaesthesia, Perioperative and Pain Medicine Unit, Melbourne Medical School, The University of Melbourne, Parkville, VIC 3010 Australia; Department of Anaesthesia, St Vincent’s Hospital, Fitzroy, VIC 3065 Australia

**Keywords:** CALD, Informed consent, Clinical research, Older people, Migrants, Language, Health literacy, Recruitment, Multimedia, Low English proficiency

## Abstract

The under-representation of culturally and linguistically diverse participants in clinical trials is an ongoing concern for medical researchers and the community. The aim of this review is to examine the complex issue of recruiting culturally and linguistically diverse (CALD) older people to medical research and to examine responses to these issues. The review focuses on (1) trends in the existing literature on barriers to and strategies for recruiting CALD and older people to clinical research, (2) issues with informed consent for CALD populations, and (3) the efficacy of innovative approaches, including approaches incorporating multimedia in research and consent processes. The literature indicates that predominant barriers to greater involvement of CALD patients in clinical trials are communication, including literacy and health literacy considerations; English language competence; and cultural factors in the research setting such as mistrust of consent processes, as well as considerable practical and logistical barriers, including mobility considerations. Some evidence exists that incorporating multimedia resources into the informed consent process can improve patient understanding and is preferred by patients, yet these findings are inconclusive. A multi-methodological approach, including the use of culturally and linguistically sensitive multimedia tools, may help address the issue of low inclusion of CALD groups in clinical research. Researcher education needs to be taken into account to address preconceptions about CALD resistance to research participation and to raise awareness of cultural concerns in regard to research participation.

## Background

Elderly people of culturally and linguistically diverse (CALD)^1^ backgrounds are under-represented in clinical research [1–3]. Under-representation of CALD patients is problematic for several reasons, but the problems centre on the decreased generalisability of trial findings and equity for patients in accessing the benefits of participating in trials. First, the generalisability of research findings is compromised [2, 4, 5], and the potential effects of variation in the pathobiology of disease, diet and lifestyle, both positive and negative, may go undetected, as do factors such as race-related differences in drug responses [3]. Some health problems are more prevalent among particular minority groups, and a disparity exists in health outcomes according to ethnicity [6] and between minorities and non-minorities [2]. Another concern is that healthcare provision is not equal to all [7, 8]; people who participate in research may receive better care and have better health outcomes [9]. The disadvantaged sectors of the population can be excluded from the potential benefits of trial participation [10], which can provide newer, higher-quality or specialised care and monitoring that is made available to participants in trials [11].

The necessity of including older people from minority groups in clinical research will intensify given that older people are a fast-growing segment of the population worldwide [12]. In 2012, in Australia, people aged 65 years and over constituted 14 % of the population, but the proportion is projected to increase to 22 % in 2061, and to 25 % in 2101 [13]. Currently, people from a CALD background make up a substantial proportion of Australia’s ageing population, with over 20 % of people older than 65 years of age having been born outside of Australia, and in just a few years’ time, by 2021, that figure will rise to 30 % [14].

### Policy promoting equitable inclusion in clinical trials

There is a call for health inequalities to be addressed, for research to focus on underrepresented groups [11, 15, 16], and for strategies to be developed to encourage the participation of these groups [7]. Over the past two decades, many studies have aimed to determine the barriers to minority groups participating in clinical research [3, 4, 17–20] and, more specifically, elderly ethnic minority groups [2, 21–23]. This is particularly notable in the United States, where legislation in 1993 has subsequently required the inclusion of women and ethnic minorities in research [24]. In Australia, no legislation explicitly requires the inclusion of these traditionally under-represented populations in clinical research, but the National Statement on Ethical Conduct in Human Research [25] states that all research should be ‘just’. This includes ensuring that, under section 1.4:

*(a)…the selection, exclusion and inclusion of research participants is fair…*

*(b) there is no unfair burden of participation in research on particular groups; …*

*(d) there is fair distribution of the benefits of participation in research* [25].

In addition, one of the primary goals of the Australian government’s *National Ageing and Aged Care Strategy for People from Culturally and Linguistically Diverse (CALD) Backgrounds* is better practice through improving research and data collection mechanisms that are inclusive of the ageing CALD population [14].

### Health literacy and ethnicity

A known correlation exists between the level of health literacy and ethnicity, with minority populations suffering from lower levels of health literacy than the general population [11]. Furthermore, people with low English proficiency (LEP) have been shown to be disadvantaged in the informed consent process, scoring lower in measures of informed consent than fluent English speakers [26]. Lower health literacy is strongly associated with less engagement with health care and, thus, poorer health outcomes [27]. Available health education materials are often not literacy-level appropriate [27–29]. In Australia, only 33 % of the overseas-born population has ‘adequate or better’ health literacy compared to 43 % of the Australian-born population, and the figures are lower for more recent arrivals (27 %) and for people whose first language is not English (26 %) [30]. Participating in a clinical trial is one health-related decision people from a CALD background may make in their lifetime, and evidence exists that the under-representation of CALD patients in clinical research is partly due to a limited awareness and understanding of research opportunities and processes [11].

### This review

Providing appropriate information to research participants is central to good clinical research practice [16, 31, 32] but presents challenges in approaching CALD patients. In cases where CALD patients do not speak English or have LEP or literacy, they are often not approached about participating. This occurs for two reasons: first, inadequate evidence is available on effective strategies for recruiting CALD patients, and second, research funding rarely covers the costs of developing CALD strategies.

While positive intent exists on the part of governments and the health sector to address the issue of low participation of CALD groups and while advances have been made in terms of identifying barriers and useful strategies associated with inclusion, minority groups are still not well represented in clinical trials [1, 9, 33]. This review examines current barriers to and strategies for involving CALD patients in clinical research, as well as the issues associated with informed-consent procedures in this group and the strategies that have been used to try to overcome these barriers. We also examine the outcomes of interventions trialling multimedia for the research-consent process in both English speakers and CALD patients in order to assess the feasibility of adapting such technologies for older CALD people.

## Methods

The key research areas under review are studies focused on the following:

- Identifying barriers and developing strategies to overcome the lack of inclusion of CALD groups in medical research
- Identifying barriers and developing strategies to overcome the lack of inclusion of older people in medical research
- Improving the informed consent process
- The use of multimedia aids in the informed consent process

We conducted a search in MEDLine in May 2015, and no results were returned for the defined topic of improving the informed consent process to improve CALD participation in clinical research utilising methods incorporating multimedia technology (e.g. computers, video, and tablets/iPads). The lack of studies trialling innovative methods of obtaining informed consent in populations where English language proficiency is low has been commented on in the past. For example, Hendrickson [34] found no documented use of videotape recruitment of non-English-speaking participants prior to her study. The dearth of existing reports examining the use of tablets in clinical trial research has also been noted [35].

In response to this finding, we then broke the research topics down into their components to be examined separately: research on the participation of CALD groups in medical research, research on the participation of older people in medical research (CALD and the elderly in general), studies on informed consent in a CALD context, and multimedia use in a research informed consent context. Combinations of the following terms were used in the search query: ‘informed consent’ AND/OR ‘migrants’, ‘minorities’, ‘low English proficiency’, ‘multimedia’, ‘elderly’, ‘iPad’, ‘video’, and ‘computer’.

## Findings

### Barriers to the inclusion of elderly CALD people in clinical trials

The major themes consistently identified by researchers as barriers to CALD participation in clinical research are: (1) mistrust; (2) communication barriers, including the complexity of written documents, language/literacy issues and lack of perceived benefit; (3) cultural barriers, including competing cultural beliefs/practices concerning health; (4) economic and time constraints; (5) mobility issues and health issues; and (6) opportunity barriers.

#### Mistrust

Mistrust is a commonly cited barrier to inclusion, manifesting in a number of ways: mistrust of mainstream society [6], of the scientific community or of research institutions [23], perhaps due to awareness of unethical practices on the part of the medical research community in the past [2, 6, 36], or a perception that academic institutions are elitist and not committed to the welfare of ethnic minority communities [17]. Alternatively, the mistrust may be directed towards the general healthcare system or be culturally generated, as for example when the medical practices of the culture of origin differ substantially to those of mainstream Western physicians [6]. Mistrust issues may be more pronounced among the elderly [37] who, because of increased frailty, can feel more vulnerable to potential exploitation [2].

#### Communication barriers

Language is an evident communication barrier in CALD groups [6]. Communication barriers also include issues associated with complex forms and informed consent procedures [38, 39], which are amplified for people with limited English language and/or low literacy [28]. Achieving true informed consent, even amongst those for whom English is a first language, can be difficult [28, 39, 40]. Much debate has occurred on the comprehensibility of participant information forms, which are often notoriously lengthy and require a high (often tertiary) level of reading comprehension [17, 41–43]. Ensuring that a patient understands to what he/she is consenting becomes even more problematic in the presence of CALD barriers. While ensuring informed consent is paramount, the process of obtaining consent may end up having the undesired effect of creating barriers by arousing suspicion, promoting mistrust, and causing misunderstanding, which may ultimately discourage participation [44].

Failing to communicate research findings with communities is also a known barrier [23]. Among the many reasons for low success in clinical trial recruitment in general is a ‘perceived lack of information about the purpose, procedures and value of clinical trials that [patients] have been asked to contribute to’ (p. 111) [45]. This issue has been highlighted in studies on the elderly [37, 46].

#### Cultural barriers

Cultural barriers to research recruitment include inappropriate information provision [10], unsuitable data collection and assessment measures [47, 48], cultural stigmas associated with illness, and a lack of social presence in the communities [1]. Specifically, for older CALD people, a lack of cultural diversity amongst research staff can act as a barrier to CALD participation; conversely ‘race matching’ (alignment of the race of the patient and researcher) may enhance participation [23, 49]. Beliefs about health and health care can vary significantly according to different cultures [8, 50] and may clash with the prevailing norms in the mainstream society in question, making individuals from CALD backgrounds less likely to explore all the healthcare options available to them.

Important cultural considerations have been identified in the consent process. Obtaining written informed consent from research participants remains standard practice [25]. However, it may be inappropriate to seek written consent from participants of certain cultures [49]. Killawi and colleagues [48] identified potential problems with obtaining signed, written consent, including an association with formal transactions, the arousal of suspicion or concern, the potential for threat for illiterate persons, or the implication of a lack of trust. Another factor undermining signed written consent is the possibility of the request for a signature being perceived as insulting if one’s word has already been given [51].

#### Economic and time constraints

CALD status and lower socioeconomic status are two factors that are often connected [9], and socioeconomic constraints have been cited by many researchers as barriers to recruitment of CALD participants in medical research [6, 10, 52, 53]. Time constraints, including those connected with family and job responsibilities, are also preventive factors in study participation [20, 53].

#### Mobility and health issues

Restrictions required by religious or cultural precepts may mean that women in some CALD groups may have compromised mobility resulting from their dependence on their husbands or another male relative for transportation [48]. For the elderly in general, additional mobility barriers such as physical frailty, increased health issues, and illness can make it difficult for this group to participate in clinical trials [22]. Common conditions such as visual impairment or decreased manual dexterity can inhibit participation; for example, written documents are more of a barrier [3, 54]. The elderly in general suffer greater degrees of social isolation, making them a less accessible group [2, 37].

#### Opportunity barriers

Barriers are also created for CALD groups by the research process. These factors are important, since they determine whether or not a person is given the opportunity to participate in research at all. For example, people with LEP or low levels of literacy are routinely excluded from many clinical trials because they are viewed as unable to provide truly informed consent [6, 41]. Most research protocols do not engage with language proficiency at all [55]. Furthermore, when clinicians believe the costs or burden of data collection are too great, they are less likely to refer or recruit patients to clinical trials [56]. In a systematic review of studies on recruitment of underrepresented groups into clinical trials, 11 studies out of 18 found clinician attitudes were a barrier to enrolment, whereas only three of these studies found clinician attitudes to be an enabler [56]. Provider attitudes that hindered patient recruitment related to patient age, comorbidities, disease stage, and/or a perception of patient mistrust of researchers.

In another review of studies examining barriers to recruitment of underrepresented populations in clinical trials, Ford et al. found that the most commonly reported barriers related to the opportunity to participate in trials [10]. Potential participants were excluded from trials because of factors such as age, socioeconomic status, ethnic minority status, and comorbid conditions. This study also identified provider attitudes and eligibility criteria as the most frequently reported barriers to participation.

### Overcoming the lack of inclusion of elderly CALD in clinical research

This section presents reported interventions and strategies to help overcome barriers impeding the inclusion of CALD groups in general, and from elderly people from a CALD background specifically, in clinical research. Strategies are presented thematically, in response to the barriers discussed above. These interventions are summarised in Table 1.

**Table 1:** Summary of the barriers to the inclusion of elderly participants who are culturally and linguistically diverse (CALD) in clinical trials and the strategies that have been developed to overcome them

Barriers	Strategies
1. Mistrust • Of society, the scientific community, and research institutions, and of the healthcare system	1. Community relationship building & outreach • Enrol community gatekeepers in the research • Build coalitions & partner with physicians • Enrol trusted and prominent community members (community and religious leaders) • Make use of other referral sources • Get involved with the community (attend events, etc.) • Provide support to the community outside of research in ways that are meaningful to the community • Maintain connections with communities • Involve the community in study design and implementation
2. Communication • Low English proficiency • Information presented is too complex • Low health literacy • Failure of researcher to communicate study rationale • Failure of researcher to communicate research findings	2. Communication – initial & ongoing • Provide education about health and medical research • Provide training for key community representatives • Provide community information sessions • Explain potential benefits of participation • Engage in follow up: provide feedback and study results to participants • Provide translations of forms and materials • Provide simple, appropriate, and systematic explanations of research • Use innovative materials to enhance comprehension (e.g. video presentations, animations). • Simplify language and decrease content in consent documentation • Apply user-friendly formatting and presentation (large graphics, both visual and audio input)
3. Cultural • Stigma • Culturally inappropriate assessment measures • Potential clash of beliefs around health care • Potential alienation of participants if staff lack cultural diversity • Potential issues with requiring signed written consent	3. Cultural sensitivity • Be informed about the background and cultural circumstances of the CALD group in question • Use culturally appropriate language, symbolic gestures, and ideas. • Respect differences • Make provision for possible adaptations based on cultural differences • Use bilingual/bicultural staff and staff reflective of the community • Ensure consent process is culturally compatible/acceptable (e.g. accept verbal consent if preferred by participant)
4. Economic & time constraints • High likelihood of socioeconomic hardship • Limited availability of participants	4. Facilitate access to research studies • Take the research to the community • Provide reimbursement for travel expenses • Provide childcare • Be flexible with scheduling • Conduct research within the community/conduct home visits • Provide transportation
5. Mobility • Mobility may be restricted for cultural or religious reasons. • In elderly populations, mobility may be a factor because of increased frailty and/or impaired functionality.	5. Awareness raising amongst researchers and other stakeholders of barriers to CALD participation • Revisit and adapt consent processes for CALD groups • Make study designs less rigid
6. Opportunity • People with low English proficiency or literacy levels are often excluded from trials. • Clinicians attitudes are a strong barrier. • If clinicians experience the costs or burden of data collection to be high, they are less likely to recruit participants.	

#### Community relationship building and outreach

Establishing and building trust is paramount in any endeavour to enrol and retain members of minority groups in clinical research. A key strategy for doing this is through developing relationships within the community of the target group [57]. This can be through establishing and maintaining a relationship with a community advisory board [3], so-called ‘gatekeepers’, such as family members or housing managers and enrolling them in the evaluation of the research opportunity, building coalitions, and partnering with physicians who provide care for the populations to be included in the trial [17, 46], thereby establishing a rapport with trusted and prominent members of the community such as community or religious leaders [6, 17, 58] and other potential referral sources [18, 57].

Becoming more involved with the community by attending events [1] and committing to support the community in ways that the community values are effective, even essential, strategies [6, 58] for building trust and demonstrating a genuine motivation to help the community. The maintenance of ongoing connections is also necessary [59], especially with community leaders [58]. This is important as a trust-building exercise in the context of ongoing research studies. Involving the community in study design and implementation [15, 22], while utilising, for example, community-based participatory research frameworks [19, 59] recognises that communities contribute knowledge to the process, which in turn helps with the formulation of recruitment strategies and encourages the involvement of minority community groups [23].

#### Communication–initial and ongoing

Providing education about the purposes of medical research is an important strategy [18]. Santoyo-Olsseen et al. [19] credit much of their success in recruiting lower socioeconomic and/or CALD adults to a diabetes risk-reduction program to their efforts to provide health education and raise awareness in the target community prior to study recruitment. Other educational strategies include providing training for key community representatives [15] or conducting community information sessions [47]. Ensuring that participants are aware of potential benefits of participation also enhances the likelihood of participation [3, 17], and making sure participants agree with the benefits to themselves and their community and communicating research outcomes have been shown to create more favourable conditions for the participation of minority groups in medical research [6, 18]. Appealing to participants’ curiosity about their own health can be a helpful engagement strategy [57]. It is important to ensure there is a process of follow-up and that participants are given feedback about or summaries of the study results [17, 18, 23, 57].

Making forms and materials available in the target group’s language(s) helps in overcoming language barriers [17]–provided the translation protocol ensures cultural and literacy-level appropriateness [29]. Explaining the research process in a simple, appropriate, and systematic way can help to alleviate concerns regarding low literacy and unfamiliarity with research [19]. Also, using innovative materials and processes such as multimedia, descriptive videos or illustrations can greatly enhance the comprehension and retention of concepts [38, 47].

Specifically, with regard to the consent process, simplifying the language and content of forms and avoiding repetition and redundancy have been identified as effective [39]; succinct, comprehensible material is preferable to overloading a patient with detail, which may simply be confusing. Stunkel and colleagues [60] found that levels of comprehension did not differ for participants who were randomly assigned to read either a ‘shorter, more readable’ consent form compared to a longer, standard consent form. Tymchuk et al. [61] found that institutionalised elderly subjects benefited from simplified versions of forms more than from either storybook presentations or videotape. Another study showed that video education increased patients’ understanding of consent information compared to oral education [62].

Formatting that presents information clearly (bullet lists, adding section breaks, signposting definitions of terms, and increasing font size for visually impaired people) is recommended as a strategy to aid comprehensibility [39]. McDougall and colleagues [1] found that their participants (older adults of ethnic and racial minorities) with visual and hearing deficits preferred PowerPoint presentations with large graphics, few words, and animation. People with hearing problems could more easily discern meanings if they could see as well as hear information. Taub and colleagues [63] confirmed the usefulness of a multi-step approach, using a comprehension test as part of the informed consent process with elderly participants.

#### Cultural sensitivity

It is important to have a clear understanding of the special circumstances of older CALD adults, which will differ according to the particular CALD group [64], including of their cultural-historical background [50] and socio-political conditions [59]. Researchers must be mindful to use culturally appropriate language [3, 6], avoid language that may be stigmatising for some groups [1], or adopt symbolic gestures where appropriate [49], and they demonstrate an appropriate attitude of openness and respect for differences that is exclusive of stereotypes, prejudices, and biases [50], while also making provision for possible adaptations based on cultural differences [65]. For example, some groups respond more favourably to personalised or direct, person-to-person recruitment strategies [19, 21, 23].

Employing researchers and staff reflective of the community, as well as employing bilingual, bicultural staff, enhances participation [6, 17, 57]. The process of gaining, building, and maintaining trust is easier with older CALD people when a researcher and respondent are from the same background [23]. This was also a key strategy identified by Sheppard and colleagues [66] in their six studies on recruiting Latinos into cancer research in the United States. They reported very high success rates for recruitment (96 % on average). Other strategies employed by this team included the following: working in Latino places of social interactions, using high-profile community leaders to disseminate information (e.g. through media), simplifying and improving comprehension in the informed consent process, and emphasising culturally important ideas such as the significance of the family in Latino culture.

Improving the consent process for CALD participants has been the focus of several studies. Some CALD groups favour verbal information over written [67] and may see verbal consent as equivalent to written consent [48]. A UK study found that obtaining informed consent from CALD persons using audio recordings was acceptable for the participants, and posed no difficulties [67]. In a linked study conducted in Bangladesh, use of audio recording for the consent process encouraged individuals with low literacy levels to participate in research [68]. Indeed, some institutions now recommend the use of audio for collecting data from minority ethnic communities [67]. Hernandez and colleagues [69] urge researchers to consider employing a waiver of signed consent of participants when conducting research in immigrant communities. Avoiding written signed consent may be more appropriate for participants with limited literacy or a lack of education, or in cases where the preferred language modality is oral. Another approach for documenting consent, which has been suggested as potentially more acceptable in given communities, is witnessed consent [47].

#### Facilitate access to research studies

In minority groups, mobility and safety concerns influence whether or not a person feels comfortable, or even able, to participate in a research study. Conducting research within a community space or offering home visits has helped with recruiting and retaining research participants [1, 3, 19, 57, 58]. Other options include taking the study to the local neighbourhood of the target group and to places where the participants live and congregate (e.g. grocery stores and recreation centres) [18]. Other strategies to improve access, including providing incentives and reimbursement (for travel/parking) [1, 6], providing transportation [19, 58], or even providing childcare [19], which can be very helpful strategies for communities where economic constraints are a barrier to research participation. However, introducing monetary incentives does raise ethical issues, as it may exert an unfair influence on some populations [70]. Flexible scheduling is an important consideration to combat the time-constraint barrier [3] and has been successfully implemented to improve enrolment and retention rates [1].

#### Awareness raising amongst researchers and other stakeholders of barriers to CALD participation

Ford and colleagues concluded that ‘Because opportunity barriers largely reflect protocol design as well as the process of study implementation, investigators play a major role in determining the extent to which trials are accessible to underrepresented groups’ (p. 238) [10].

Doctors, researchers, and ethics committees have a crucial role to play in increasing the participation of CALD groups in clinical research, while still adequately protecting participants [25, 31]. Informed consent processes need to be revisited and adapted for CALD groups. It has been suggested that ‘less rigid study designs’ (p. 238) and systems that facilitate the participation of healthcare providers are ways to create conditions that will improve the recruitment of underrepresented populations to trials [10]. Modified consent processes need to be developed to meet the needs of participants for comprehensibility and ease of use, while retaining ethical rigour. Nesting trials of innovative methodologies aimed at increasing recruitment within existing studies has been suggested as an effective way to test the robustness of new methods [71].

### The use of multimedia to improve CALD participation in research

Researchers have attributed success in the recruitment of minority groups to a multifactorial methodology [20] and to the use of ‘an array of recruitment strategies’ [9]. One problem is that engaging CALD communities requires significant resources, not the least of which is financial, that are rarely available in current research structures. Incorporating multimedia technology may be less costly in the long run and more sustainable than current practices that require interpreters. An electronic resource can be customisable to accommodate multiple languages and can be developed incorporating principles of cultural sensitivity. In addition, multimedia could be used by researchers who do not necessarily speak the language of the participant.

Furthermore, multimedia resources may have key roles to play in addressing health research literacy by explaining medical research, enabling researchers to assess comprehension through testing, and improving CALD patient comprehension of consent forms and procedures [38]. In addition, technology offers possibilities to overcome lengthy and burdensome patient information sheets, a known source of patient dissatisfaction [71]. The findings from trials of electronic resources to improve consent processes, even if only with non-CALD participants, can inform the development of resources for CALD groups.

Methods combining textual, audio, and graphic (static and/or dynamic) modes are increasingly being used in many settings. Simultaneously combining more than one mode (e.g. text and graphics) is associated with increased comprehension, learning, and retention than text alone, and splitting incoming information across more than one cognitive channel (e.g. audio and visual) is associated with significant learning gains [72].

Some reviews of clinical research using multimedia to enhance understanding of information and consent forms have reported inconsistent or minimal evidence of improvements using newer technologies [40, 45, 73], and proposed benefits to the willingness to participate and participant satisfaction have been either inconclusive or not apparent [73]. Conclusions have been limited due to novelty, inconsistent methodologies, and definitions [42]. In spite of these limitations, favourable conclusions concerning the effectiveness of multimedia in the informed consent process have also been drawn. Improved outcomes for participant comprehension [41, 74, 75], for both immediate information recall [76, 77] and delayed recall [78], participant satisfaction (particularly regarding understanding) [41, 79, 80], and reduced patient anxiety levels [78, 79] have all been attested in both systematic reviews and individual studies of multimedia approaches to consent procedures conducted with fluent English speakers. In addition, when consulted, participants have indicated a preference for incorporating multimedia in consent protocols [42].

In individual studies on consent procedures where the only difference was the medium used, patients generally preferred electronic over paper-based questionnaires in spite of the fact that the former took longer to complete [81, 82], perhaps because they had to be filled out completely in order for patients to finish them (which is in itself advantageous from a researcher perspective).

A few studies adopting interactive approaches, for example, where participants are required to demonstrate their understanding by answering a quiz after watching a video on an iPad or computer, show particular promise for improving the comprehension and retention of knowledge regarding research in participants [54, 83] and increasing participant interest in taking part in a study [76].

### Acceptability of an electronic tablet resource to older CALD people

Some questions exist concerning the acceptability of using an electronic resource with older people. Nonetheless, one study comparing the use of a tablet-based screening procedure with an integrated voice response system for recruitment and screening of patients into pragmatic clinical trials concluded that the tablet was ‘highly accepted by older patients’ [35]. Shneerson et al. [45] designed a questionnaire to elicit views from older cancer patients, their carers, friends, and families on the provision and design of multimedia resources used to deliver clinical trials information. Most respondents, whose average age was over 60, indicated that seeing a multimedia resource might encourage them to find out more about clinical trials, and more than half preferred a ‘more practical learning mode, as could be delivered via multimedia resources’. Preferred devices were mobile phones, laptops, and DVD players, although Shneerson concluded that ‘from a practical perspective tablet technologies would appear to be the most suitable delivery medium, being portable, cheap, less prone to operating system errors and simple to use’, and felt that the transition from PCs (with which they were familiar) to tablets would be relatively easy with suitable training and support.

A study evaluating user response to a talking touchscreen, introduced as a means to improve health literacy in underserved U.S. populations, found that most users, the majority of whom were computer-naïve, rated the design of the screen highly and found it easy to use [84]. It is important to take into account, nonetheless, that in some cases, there may be a correlation between difficulty filling out a questionnaire on a tablet and CALD background, increased age, lower levels of education, and comorbid medical conditions [85]. Furthermore, research into barriers to participation underlines the importance, for participants, of a human connection [21].

## Conclusions

A main finding of this review is that addressing the obstacles to CALD participation in clinical research requires a complex, multi-methodological approach. A further aid to help eliminate barriers, we believe, lies in incorporating multimedia tools in this approach. To be effective, such tools need to form part of a culturally sensitive system, which we propose would include providing resources in the preferred languages of potential participants, and tailoring them to target communities’ language, literacy, and cultural needs. We envisage that it would always be preferable to have a researcher present with a participant, both to help with using the device, and also to be a tangible human presence.

Nonetheless, another important finding of this review has been to emphasize the fact that the ultimate success of any initiatives in the area of CALD inclusion in clinical research is contingent on the willingness of stakeholder groups, and notably ethics review boards, to participate in and co-operate with the creation of novel processes or the adaptation of existing processes, especially concerning consent. This would create the conditions needed to make the participation of CALD groups in clinical research a reality. We recommend that future work in the area focus on this key issue.

## Abbreviations

CALD, culturally and linguistically diverse; LEP, low English proficiency.

## Footnotes

1 The term CALD is used in the current study. However, the term is used interchangeably with ‘migrant’ or ‘ethnic’ in the context of comparisons with other studies.
